# A review of *Siraitia grosvenorii*: Applications, breeding, and biosynthesis of mogrosides

**DOI:** 10.1016/j.chmed.2025.11.005

**Published:** 2025-11-15

**Authors:** Zuliang Luo, Yimei Zang, Jiaxian Su, Qi Tang, Limei Pan, Xiaojun Ma, Chongnan Wang, Changming Mo

**Affiliations:** a State Key Laboratory for Quality Ensurance and Sustainable Use of Dao-di Herbs, Institute of Medicinal Plant Development, Chinese Academy of Medical Sciences and Peking Union Medical College, Beijing 100193, China; b Yuelushan Laboratory, Changsha 410128, China; c College of Horticulture, National Research Center of Engineering Technology for Utilization of Botanical Functional Ingredients, Hunan Agricultural University, Changsha 410128, China; d Guangxi Key Laboratory of for High-Quality Formation and Utilization of Dao-Di Herbs, Guangxi Botanical Garden of Medicinal Plants, Nanning 530023, China; e Guangxi Crop Genetic Improvement and Biotechnology Laboratory, Guangxi Academy of Agricultural Sciences, Nanning 530007, China

**Keywords:** biosynthesis, food-medicine homologous, mogrosides, mogroside V, *Siraitia grosvenorii* (Swingle) C. Jeffrey ex A. M. Lu et Z. Y. Zhang

## Abstract

*Siraitia grosvenorii* is a premier food-medicine homologous species recognised by China’s National Health Commission and produces mogrosides as its primary active component. These compounds exhibit biological activities, including the regulation of blood sugar, fat metabolism, and immune function regulation. They are classified as high-intensity, non-nutritive sweeteners with significant medicinal potential and nutritional value. This review systematically explores the applications of *S. grosvenorii* in traditional medicine and foods, with a focus on advances in the conservation of germplasm resources and the traditional breeding of elite varieties, mogroside biosynthetic pathways and the characterization of key genes, and synthetic biology platforms for mogroside production. We have identified the low content of mogroside V in *S. grosvenorii* as the main reason for its limited application. To address this issue, we propose two strategic approaches: enhancing mogroside content *in vivo* through molecular design breeding and developing three synthetic biology platforms for mogrosides synthesis to increase yields. These solutions offer viable ways to reduce production costs and expand the commercial use of *S. grosvenorii* medicines and sweeteners.

## 1 Introduction

*Siraitia grosvenorii* (Swingle) C. Jeffrey ex A. M. Lu et Z. Y. Zhang is an herbaceous perennial plant native to the southern regions of China, primarily found in subtropical areas such as Guangxi, Guizhou, and Hunan Provinces. The fruit of *S. grosvenorii* is among the first medicinal materials recognized by China’s National Health Commission as a food-medicine homologous resource. Historical records indicate that *S. grosvenorii* has been used medicinally for over three hundred years in China (Murata et al., 2010). Traditional medical research highlights its benefits, including clearing heat, relieving cough, resolving phlegm, moistening the lungs, quenching thirst, and promoting bowel movements. Due to its cool nature (as defined in traditional Chinese medicine, TCM), *S. grosvenorii* is particularly effective in treating bronchitis, sore throat, and phlegm (Li & Zhang, 2000). Modern pharmacological studies have also demonstrated that *S. grosvenorii* possesses antioxidant, anti-inflammatory, anti-tumor, hypoglycemic, and hepatoprotective activities (Huang et al., 2024, Suri et al., 2021, Chen et al., 2024, Zhang et al., 2024).

The components separation and identification of *S. grosvenorii* have revealed that it contains a rich variety of active components, including triterpenoids, flavonoids, amino acids, volatiles, polysaccharides, and vitamins (Liao et al., 2008; Li and Jian, 2007, Matsumoto et al., 2009). Currently, over 70 types of mogrosides (cucurbitane-type triterpene glycosides) have been isolated and identified from *S. grosvenorii*, among which mogroside IV, mogroside V, and siamenoside I exhibit high sweetness, non-nutritive, and non-toxicity properties (Ma & Mo, 2024). Mogrosides are natural non-nutritive substances known for their intense sweetness. They are primarily composed of mogroside V, mogroside IV and siamenoside I. Their sweetness is approximately 300 times that of 5% sucrose (Kasai et al., 1989). In recent years, mogrosides have gained widespread application in the food additive and health food industries (Pawar et al., 2013, Pandey and Chauhan, 2020). In the 1990s, the US Food and Drug Administration (FDA) approved mogrosides as a sweetener for food. Similarly, China approved mogrosides as a sugar substitute for obese and diabetic patients in 1996, allowing them to partially or completely replace sucrose in the health food industry (Hu & Ma, 2008). Modern pharmacological studies have demonstrated that mogrosides possess multiple biological activities, including regulating blood sugar balance, fat metabolism, and regulating immune function. This makes mogrosides one of the few new sweeteners derived from TCM with therapeutic functions (Xu et al., 2013, Pan et al., 2009, Shi et al., 2014). Consequently, mogrosides exhibit significant potential for application in food additives, functional foods, and TCM products with both dietary and medicinal values.

Although *S. grosvenorii* exhibits excellent pharmacological effects and holds significant market potential as a new type of sweetener, the primary method for obtaining mogrosides still relies on plant extraction (Li et al., 2022). *S. grosvenorii* displays distinct periodic, regional, and seasonal characteristics in planting and harvesting. The limited suitable cultivation area, along with challenges such as the necessity for artificial pollination, restricts the scale of *S. grosvenorii* cultivation (Tu et al., 2017). Furthermore, the content of mogroside V in *S. grosvenorii* is relatively low, averaging only 0.3% to 0.4% in fresh fruit, leading to high extraction costs. Currently, the raw materials for mogrosides on the market mainly consist of extracts containing 50% mogroside V, which are expensive (Muñoz-Labrador et al., 2021). Consequently, the development and utilization of mogrosides are significantly constrained, impeding their competitiveness with sucrose, xylitol, and stevia in the food, health product, and daily health product sectors (Ma & Mo, 2024). Therefore, increasing the content of mogrosides in *S. grosvenorii* and establishing heterologous synthesis pathways for mogrosides have become popular research directions.

With the release of extensive gene information for *S. grosvenorii*, particularly subsequent to the completion of its whole-genome sequencing, research in the functional genomics of this species has advanced rapidly (Xia et al., 2018, Tang et al., 2011, Itkin et al., 2016). Researchers, both domestically and internationally, have been actively engaged in cloning and functional studies of key enzyme genes involved in the synthesis of mogrosides. The biosynthetic pathway of mogroside V has been fully elucidated, thus laying the foundation for further research in synthetic biology (Itkin et al., 2016). Currently, the biosynthesis of mogrosides has emerged as a research hotspot, and significant progress in this area is anticipated to provide effective solutions for mogroside sourcing.

This article systematically reviews the overall status and application prospects of *S. grosvenorii* as both a TCM and a natural sweetener. It focuses on the biosynthetic pathways of mogrosides, the cloning and characterization of functional genes, and progress in synthetic biology research. This review aims to provide scientific references for the development and utilization of *S. grosvenorii* and its active component, mogrosides.

## 2 Application in TCM

*S. grosvenorii* is a unique plant in China and one of the first plants approved by the National Health Commission as a food-medicine homologous plant. It has a cultivation history of over 300 years in the field of TCM, particularly in southern China (Zhang, Huang, Wu, & Pi, 2017). *S. grosvenorii* was included in the *Chinese Pharmacopoeia* in 1977 and has been documented in subsequent editions. The *Chinese Pharmacopoeia* states that *S. grosvenorii* has a sweet taste and cool nature, is associated with the lung and large intestine meridians, and possesses functions such as clearing heat, moistening the lungs, soothing the throat, and promoting bowel movements. It is primarily used to alleviate symptoms like dry cough casued by lung heat, sore throat, and constipation due to intestinal dryness (Chinese Pharmacopoeia Commission, 2020).

**Fig. 1:**
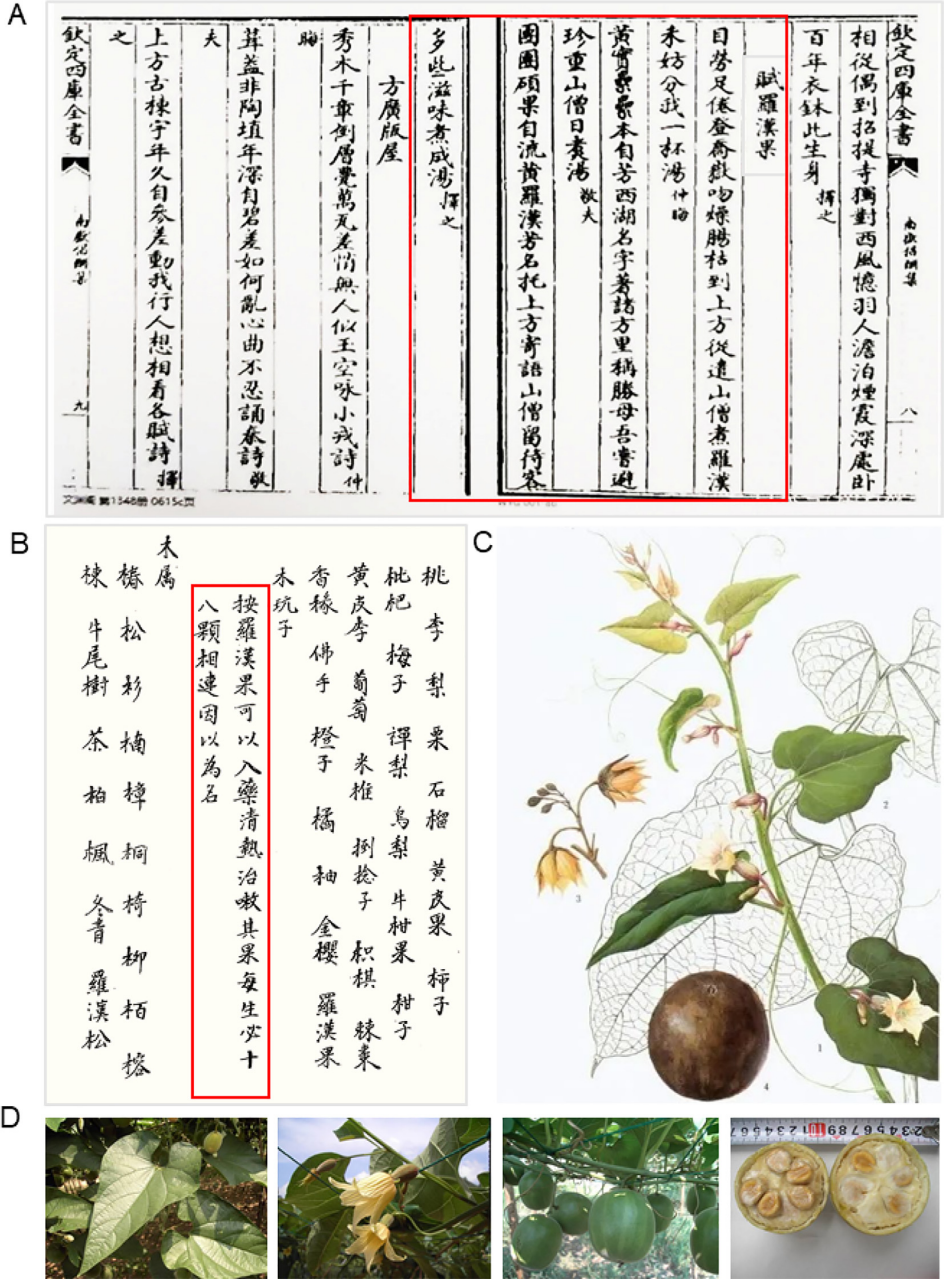
Historical documentation and botanical characterisation of *S. grosvenorii*. (A) *Siku Quanshu* records the use of *S. grosvenorii*. (B) *Xiu Ren County Chronicle* documents its medicinal effects. (C) Line drawing of *S. grosvenorii* (Chen, 2023). (D) Pictures of the leaves, flowers, fruit and seeds of *S. grosvenorii*.

*S. grosvenorii* has a documented history of culinary and medicinal use. It originated with Buddhist monks, who brewed the fruit of the plant as a herbal tea for guests. The earliest known record dates back to the Southern Song Dynasty, when the scholars Zhang Shi, Zhu Xi, and Lin Yongzhong wrote a series of poems called “*Ode to Luohanguo*” after drinking the tea at Fangguang Temple. These poems were subsequently preserved in the Qing Dynasty encyclopedia *Qin Ding Siku Quanshu-Nanyue Chang Chou Ji* (*Siku Quanshu*), constituting the first historical attestation of the plant (Fig. 1A). The medicinal properties of *S. grosvenorii* were first formally documented in the Qing Dynasty *Xiu Ren County Chronicle*, which described its use in “clearing heat and relieving cough” (Li et al., 2020a) (Fig. 1B). Subsequent records appear in various regional county annals including the “*Yongning County Chronicle*” and “*Linggui County Chronicle*” (Zeng, 2009). Further descriptions appear in the Republican-era “*Lingnan Medicinal Herb Collection*” (1936), which details its morphology and recommends preparing it with pork to treat phlegm and coughs (Ma & Mo, 2024). By the Qing Dynasty, the plant’s botanical characteristics and therapeutic applications were well established in local literature. In modern times, the morphological traits (Fig. 1C, D) and pharmacological functions of *S. grosvenorii* have been systematically standardized and authoritatively documented in the *Chinese Pharmacopoeia* and contemporary scientific research.

*S. grosvenorii* is widely utilized in the medicinal practices of ethnic minorities such as the Zhuang, Yao, Dong, and Miao, exhibiting medicinal effects like those found in TCM. It is included in the *Quality Standards for Zhuang Medicines in Guangxi* (2008 edition), where it is recognized for its abilities to “clear the airways, detoxify, stop coughs, resolve phlegm, and promote fluid production and bowel movements”. The *Yao Ethnic Medicinals in China* records that *S. grosvenorii* is sweet taste and cool nature, possessing functions such as clearing heat, relieving summer heat, stopping coughs, expelling phlegm, moistening the intestines, and cooling the blood. It is used to treat symptoms including summer heat-induced thirst, dry cough, bronchitis, tuberculosis, colds, throat pain, constipation due to intestinal dryness, and diabetes (Qin, Luo, & Gao, 2002). The *Compilation of Dong Cultural Heritage* (Volume 3), includes a section on Dong medicine, stating that *S. grosvenorii* is “sweet in taste, cool in nature, and has the functions of clearing heat, moistening the lungs, and promoting bowel movements” (Lu & Wu, 2006).

Modern pharmacological studies indicate that mogrosides possess a variety of effects, including cough suppression, phlegm clearance, antioxidant properties, blood sugar reduction, anti-tumor and anti-inflammatory effects, hepatoprotective liver-protective activities, and improvement of physiological functions (Chen et al., 2024). Mogroside V can significantly reduce the frequency of coughs in mice, prolong the cough latency period, and alleviate bronchial spasms (Liu et al., 2007; Wang et al., 1999). Mogrosides act as Toll-like receptor 4 (TLR4) receptor agonists, which may alleviate diseases such as pulmonary fibrosis, allergic asthma, and lung inflammation (Dong, Lu, Yan, & Li, 2022). They exhibit both preventive and therapeutic effects on acute liver injury in mice and chronic liver injury in rats, along with certain anti-fibrotic effects on the liver (Xiao and Wang, 2013, Wang et al., 2013). The water extract of *S. grosvenorii* can lower fasting and postprandial blood sugar levels in type 1 diabetic mice, improving the degree of pancreatic lesions. The blood sugar-lowering effect is primarily attributed to mogrosides, which do not affect the weight, blood sugar, or glucose tolerance of normal mice (Suzuki et al., 2005, Qi et al., 2003, Chen et al., 2006). A recent study examined the regulatory effects of mogroside V on glucose, lipid, and protein metabolism in type 2 diabetic mice. The findings revealed that mogroside V effectively alleviates metabolic dysfunction through multiple pathways, including activation of the mammalian target of rapamycin (mTOR)/phosphorylated ribosomal protein S6 kinase 1 (p-P70S6K) signaling pathway, upregulation of glycolytic enzymes, and enhancement of insulin sensitivity (Huang et al., 2025). Additionally, the water extract of *S. grosvenorii* enhances the pancreas’s ability to secrete insulin in a fasting state, resists oxidative stress caused by diabetes, and slows down kidney damage induced by diabetes (Suzuki et al., 2007). These studies demonstrate that the modern efficacy research on *S. grosvenorii* is consistent with the clinical applications recorded in ancient texts and materia medica.

Emerging evidence from the past decade reveals novel pharmacological activities of *S. grosvenorii*, including anti-depression-like effects, anti-fatigue effects, anti-schizophrenia effects, anti-Parkinson’s disease effects, anti-fibrosis effects, and inhibition of lipid deposition and obesity. In neurological disorders, mogroside V demonstrates antidepressant effects in CUMS-induced depressive mice by modulating inflammatory/oxidative stress pathways and the brain-derived neurotrophic factor (BDNF)/tropomyosin receptor kinase B (TrkB)/protein kinase B (AKT) cascade, while also ameliorating schizophrenia-like behaviors in MK801-treated models (Liu et al., 2023, Ju et al., 2020). Mogrol has been shown to exert anti-fatigue effects by augmenting the relative protein expression of phosphorylated AMP-activated protein kinase alpha (p-AMPK*α*), AMPK*α*, peroxisome proliferator-activated receptor gamma coactivator −1*α* (PGC-1*α*), and mitochondrial transcription factor A (TFAM) within the skeletal muscle of fatigued rats (Guo and Wen, 2022). For Parkinson’s disease (PD), mogroside V and mogrol protect dopaminergic neurons via mitochondrial function restoration and SIRT3/SOD2 regulation in both MPTP/rotenone-induced PD mice and SH-SY5Y cells (Tang et al., 2024, Luo et al., 2022). Anti-fibrotic effects are mediated through mogroside IIIE’s inhibition of TLR4/MyD88-AMPK/NF-*κ*B pathways in lung/liver fibrosis, outperforming prednisone in pulmonary models, whereas mogrol attenuates TGF-*β*1/Smad2/3-driven fibrogenesis (Tao et al., 2017, Shi et al., 2023; Liu et al., 2021a). Mogroside V restores lipid homeostasis by activating AMPK, which regulates SREBP1, PPAR-*γ*, and PPAR-*α* to balance lipid synthesis and breakdown, effectively reducing high-fat diet-induced liver steatosis in mice. Additionally, nanoselenium-enriched *S. grosvenorii* modulates gut microbiota and improves metabolic function by preserving liver lysine metabolism while suppressing fatty acid *β*-oxidation, offering anti-obesity benefits (Wang et al., 2023). These findings underscore *S. grosvenorii*’s multifaceted therapeutic potential across neuropsychiatric, fibrotic, and metabolic diseases.

**Fig. 2:**
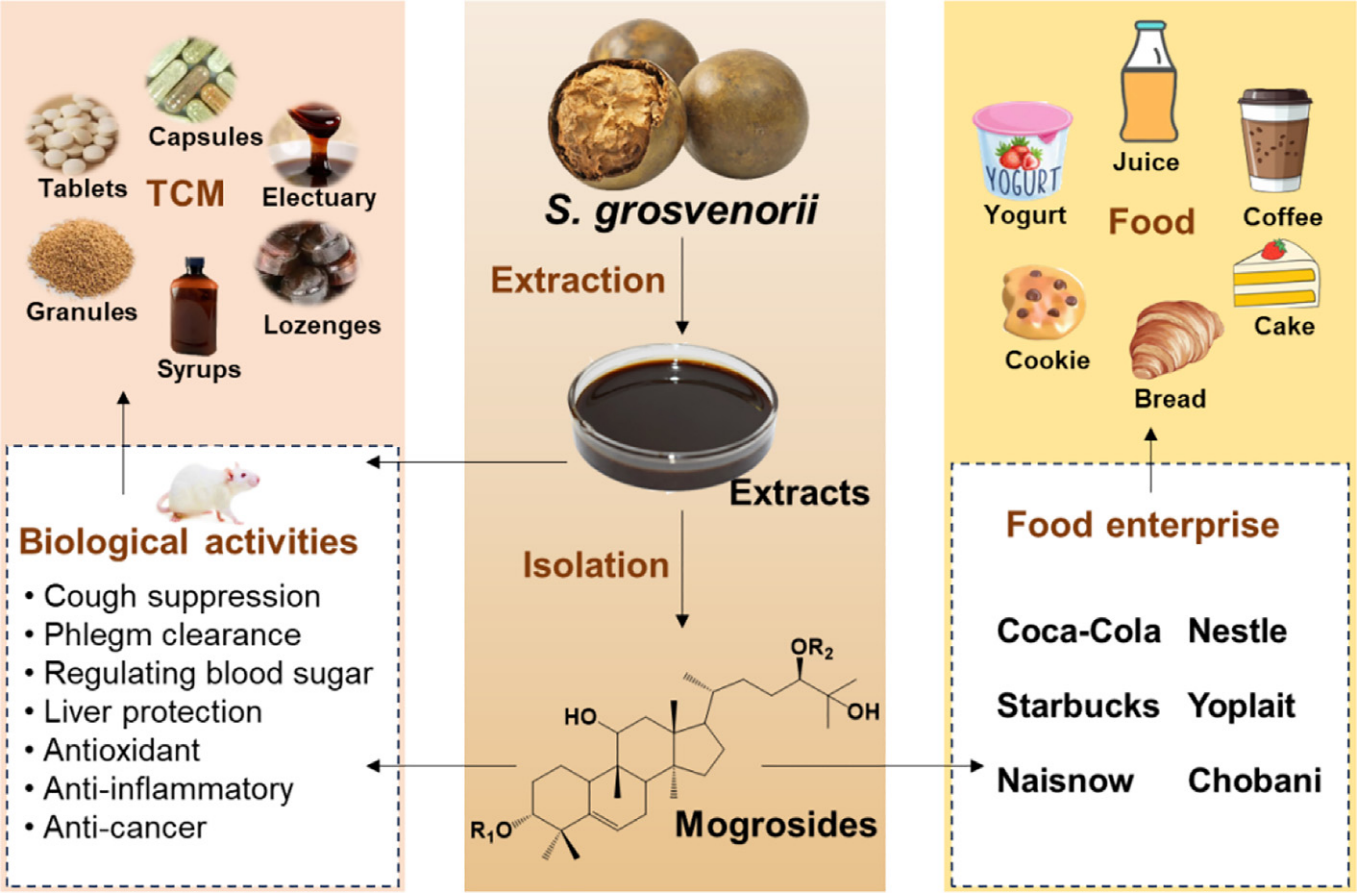
Applications of *S. grosvenorii* in TCM and food field.

*S. grosvenorii* has excellent medicinal properties, with mogrosides as its main active ingredient. In addition to being used as a TCM, it is also produced in various forms such as granules, syrups, tablets, and capsules, with dozens of production enterprises involved (Ma & Mo, 2024) (Fig. 2). The related TCM products include two main categories: heat-clearing and detoxifying products, and cough-relieving and phlegm-expelling products (Table 1). However, the significant effects of *S. grosvenorii* such as blood sugar reduction, antioxidant activity, anti-inflammatory properties, anti-tumor effects, hepatoprotective activities, and bowel movement promotion have not been well developed and utilized, indicating a need for further research and development of related products.

**Table 1:** Main TCM products containing *S. grosvenorii* extracts and production enterprises.

No.	Product names	Efficacies	Number of production enterprises
1	Compound monk fruit cough granule	Clearing heat and relieving the lungs, calming cough and expelling phlegm.	40
2	Monk fruit and yuzhu granules	Nourishing YIN and moistening the lung, relieving cough and promoting fluid production.	10
3	Compound monk fruit lung-clearing granules	Clearing heat and transforming phlegm, moistening the lungs and stopping cough.	1
4	Monk fruit and chrysanthemum granules	Clearing heat and moistening the lungs, stopping cough and brightening the eyes.	1
5	Monk fruit cough syrup	Expelling phlegm and stopping cough.	24
6	Compound monk fruit lung-clearing syrup	Clearing heat and transforming phlegm, moistening the lungs and stopping cough.	1
7	Compound monk fruit lozenges	Nourishing YIN and moistening the lung, reducing swelling and benefiting the throat.	1
8	Watermelon frost throat l ozenges	Clearing heat and detoxifying, reducing swelling and benefiting the throat.	1
9	Jinsangzi throat tablets	clearing heat, detoxifying, and benefiting the throat.	1
10	Compound monk fruit cough tablets	Expelling phlegm and stopping cough.	1
11	Monk fruit cough tablets	Expelling phlegm and stopping cough.	1
12	Monk fruit cough capsules	Expelling phlegm and stopping cough.	5
13	Monk fruit and yuzhu powder	Nourishing YIN and moistening the lung, relieving cough and promoting fluid production.	2

## 3 Application in the food field

Mogrosides, as natural, non-nutritive, functional high-intensity sweeteners, are the best sweetener for individuals with obesity, hypertension, diabetes, and heart disease who require low-calorie foods and cannot consume sucrose. They are widely applied in health food products (Fig. 2). The sweetness levels of mogroside IV, mogroside V, siamenoside I, and isomogroside V are 300 times, 378 times, 465 times, and 500 times as sweet as 5% sucrose, respectively (Murata et al., 2006). Due to their high sweetness, zero calories, non-toxicity, absence of aftertaste, good solubility, and significant medicinal effects, mogrosides have become one of the few natural, non-nutritive sweeteners derived from TCM with therapeutic functions, suitable for consumption by people with diabetes and obesity (Marone, Borzelleca, Merkel, Heimbach, & Kennepohl, 2008). In China and Japan, mogrosides extracts have been approved as healthy sugar substitutes for individuals with obesity and diabetes (Gong et al., 2019).

Mogrosides extract can be consumed as a health-promoting juice or food additive and can also be used to prepare sugar-free foods (Yang, Guo, Zhang, Yang, & Zhang, 2016). In 1995, the Food Safety Commission of Japan (FSCJ) first approved mogrosides as a food additive, making Japan one of the first countries to approve mogrosides as sweeteners for food and beverages (Liu et al., 2019). In China, the National Food Additives Committee approved mogroside extract as a food additive in 1996, allowing it to partially or fully replace sucrose. The formal implementation of the *Mogrosides Food Additive* regulation in 2017 further clarified its application in the Chinese market (Chen et al., 2022b). Moreover, in the latest version of the *National Standard for Food Safety: Standards for the Use of Food Additives* (GB 2760–2024), mogrosides can be added to various foods with no restrictions on the maximum usage level. In 2009, the U.S. Food and Drug Administration (FDA) began recognizing a series of mogrosides as Generally Recognized as Safe (GRAS) (Harshita, 2023). Mogrosides from BioVittoria (Hamilton, New Zealand) and Guilin Layn Natural Ingredients (Guangxi, China) received GRAS certification from the FDA in 2010 and 2011, respectively, successfully entering the U.S. market (Pawar, Krynitsky, & Rader, 2013). This means that mogrosides can be legally used as sweeteners in foods and beverages in the U.S. In 2010, the European Union approved mogroside extract as a food additive through the *Regulation of the European Parliament and Council on Food Additives* [Regulation (EU) No. 1129/2011]. This regulation specifies the categories of food in which mogrosides can be used and their maximum usage levels (Chen et al., 2024). Currently, over 20 countries, including China, the U.S., and Japan, allow the use of mogrosides as food additives (Chen et al., 2022b). With growing consumer awareness of healthy sweeteners, the demand for mogrosides continues to grow, making it one of the fastest-growing herbal extracts. The future development potential for mogrosides market, both domestically and internationally, is immense.

Due to the sweetness of mogrosides being hundreds of times sweeter than sucrose and having zero calorie characteristics, they are often used in the food industry as substitutes for aspartame, sodium saccharin, and other sweeteners (Castro-Muñoz et al., 2022). In the food industry, mogrosides extract has been used to produce various consumables, including low-sugar beverages, *S. grosvenorii* composite fruit drinks, *S. grosvenorii* tea, *S. grosvenorii* fermented wine, *S. grosvenorii* candies, dairy products like yogurt and ice cream, as well as baked goods such as cakes, cookies, and bread (Gong et al., 2019). Internationally, mogrosides are important ingredients in the sugar-free products of over 20 well-known large companies, including Nestlé, PepsiCo, Coca-Cola, Uni-President, General Mills, Kellogg’s, and Chobani (Ma & Mo, 2024). Currently, more than 5 000 products using mogrosides have been launched in the global market (Ma & Mo, 2024), including Starbucks double shot coffee and protein drinks (coffee, dark chocolate, vanilla bean), Nestlé's fat-free frozen coffee (latte, cappuccino, mocha latte), Hubert lemonade, Chobani's Simply 100 series, Yoplait yogurt, and fruit juices, among others.

In China, mogrosides are used in various low-sugar beverages and health foods. The well-known domestic tea beverage company, Naisnow, began using mogrosides to replace synthetic sweeteners like sucralose in 2020. The sugar company Swire Sugars also uses mogrosides as sugar substitutes. Additionally, *S. grosvenorii* is used in many health products, including throat lozenges, cooling throat lozenges, *S. grosvenorii* and honeysuckle lozenges, *S. grosvenorii* osmanthus tea, *S. grosvenorii* ginseng tea, *S. grosvenorii* juice powder, *S. grosvenorii* turtle jelly, *S. grosvenorii* honey, and *S. grosvenorii* puree (Dong, Lu, Yan, & Li, 2022). A compound beverage using *S. grosvenorii* and *Prunella vulgaris* as the main ingredients has been developed, resulting in a flavorful drink with a sweet taste that offers heat-clearing and spleen-nourishing effects (Wei et al., 2020). Additionally, a health complex drink made from sea buckthorn leaves and *S. grosvenorii* has been created, this drink is low in caffeine and sugar and contains flavonoids (Ou, 2017). Low-sugar milk tea and chewing gum products with mogrosides as a sweetener are also being produced (Xiong et al., 2021, Zhou et al., 2016). The combination of erythritol, mogroside, and stevia glycoside in a ratio of 49.5:1.0:1.0 serves as a sucrose substitute, meeting the demand for low-sugar and low-calorie yogurt varieties (Zhu, Hu, Huo, & Mi, 2022). Furthermore, mogrosides are gradually being applied to dairy beverages, enhancing their health benefits by improving flavor, reducing sugar and fat content, and increasing nutritional value (Liu & Zhang, 2021).

*S. grosvenorii* has a long history of use in food and medicine, with significant advancements in its cultivation, TCM, and development of sweeteners and health products. This has formed a complete industrial chain that has contributed to local prosperity. However, the industry is still in its infancy due to ongoing constraints. Mogrosides, the key bioactive compounds in *S. grosvenorii*, serve dual roles as antitussive agents and high-potency natural sweeteners. Despite their value, challenges persist: the fruit naturally contains low concentrations of mogrosides due to its seed-dense structure (seeds lack mogrosides), which results in the inefficient utilization of raw materials during extraction. This elevates production costs, with raw materials accounting for around 70% of total expenses. Ultimately, this leads to high market prices that hinder industry expansion. Current research predominantly focuses on mogroside extraction, analytical methods, toxicological and pharmacological properties, and applications. While extraction yields have reached relatively high levels, there is limited scope for further cost reduction through process optimization.

In order to drive market growth and industrial scaling, it is imperative to find innovative ways to reduce production costs. Two promising areas of research have emerged: (1) the use of synthetic biology for mogroside fermentation on an industrial scale. (2) Genetic breeding of *S. grosvenorii*. The subsequent chapters will present the practical achievements of our research group in the genetic breeding of *S. grosvenorii*, and discuss the rapid advancements in synthetic biology of mogrosides.

## 4 Germplasm resources and breeding of *S. Grosvenorii*

The primary objectives of *S. grosvenorii* breeding focus on enhancing yield and increasing the content of mogroside V. Our research team has systematically collected and identified germplasm resources and male pollinator varieties. We have also implemented conservation measures and varietal registration for obsolete cultivars that were previously abandoned in commercial production. Over the past decade, we have successfully developed innovative breeding strategies, including sexual hybrid breeding and polyploid breeding. These strategies have enabled us to select first-generation cultivars such as “Yongqing No.1” and “Yongqing No.2”, as well as the development of widely adopted commercial varieties, including “Bolin No.1”, “Bolin No.2” and “Bolin No.3”, as well as “Dadi No.1” and “Dadi No.2”. Furthermore, we have pioneered the development of second-generation cultivars, as demonstrated by “Yaoxiong No.1” and “Yaoyuan Seedless No.1”. In addition, we have expanded our research scope to encompass critical supporting technologies for advanced breeding programmes. Systematic research has been conducted into germplasm conservation, polyploid induction and parthenocarpy induction methodologies. These technological advancements will be explained in more detail in subsequent sections (Ma & Mo, 2024).

### 4.1 Investigation, collection and evaluation of germplasm resources

The genuine producing areas of *S. grosvenorii* are located in the Yongfu and Lingui counties of Guangxi Province. In recent years, however, limited cultivation has spread to neighboring provinces, including Hunan, Fujian, Guizhou, Jiangxi, Guangdong, Sichuan and Yunnan. A survey of wild and cultivated germplasm resources was conducted in 2008 and 2021 across thirteen counties in Guangxi and fifty locations in eight provinces (Hunan, Guangdong, Guizhou, Jiangxi, Yunnan, Sichuan, Fujian and Hainan). It was revealed that wild germplasm has become critically scarce due to overharvesting, habitat destruction and pest infestations, sporadically distributed in Guangxi, Hunan, Jiangxi, Guangdong and Fujian (Mo et al., 2023). Cultivated germplasm has also suffered dramatic reductions, driven by the phasing out of varieties of inferior fruit quality and the excessive promotion of elite tissue-culture cultivars through monoculture. Before the 1990s a variety of landraces were cultivated, such as Qingpiguo, Hongmaoguo, Baimaoguo, Dongguahan, Changtanguo, Lajiangguo, Chashanguo, Malinguo and Diyouzi. By 2005, Qingpiguo dominated cultivation, while Hongmaoguo, Baimaoguo, Dongguahan, Lajiangguo and Chashanguo were cultivated only in limited quantities (Li, Jiang, & Li, 2010). Currently, the monoculture of the Qingpiguo variety prevails, with other landraces having been nearly eradicated due to deficiencies in productivity, inferior quality, unfavorable fruit morphology or excessive vegetative growth.

Most cultivated germplasm now exists solely as *in vitro* preservations within specialized research institutions and production enterprises. However, ongoing losses persist due to inadequate preservation infrastructure, suboptimal methodologies and unstable funding. There is an urgent need to implement integrated conservation measures, including the establishment of protected natural reserves, germplasm nurseries, and advanced germplasm banks.

### 4.2 Germplasm conservation techniques in S. grosvenorii

Due to the severe threats from nematode infestations and viral diseases in field cultivation, the conservation of *S. grosvenorii* germplasm primarily relies on *in vitro* tissue culture methods. Our group has established a stable, long-term tissue culture preservation protocol by systematically optimizing the composition of the medium, the selection of the explants, the culture temperature and the application of growth retardants (Fu, 2005, Liu, 2009).

### 4.3 Hybrid breeding of elite S. grosvenorii varieties

#### 4.3.1 Hybrid selection of first-generation seeded diploid varieties

Natural *S. grosvenorii* cultivars exhibit suboptimal comprehensive traits. However, significant segregation in the F_1_ hybrid progeny, particularly in target traits such as fruit size, shape, and color, provides opportunities for genetic improvement through hybridization.

To illustrate this point, the breeding of the large-fruit variety “Yongqing No. 1” will be taken as an example. In order to address the low proportion of large fruits in existing cultivars, our research team conducted a systematic collection of germplasm in September 2004. A Qingpiguo landrace was acquired from Longjiang Township, Yongfu County, and fully matured fruits were selected as the maternal parent (designated 1B). Seeds from these fruits were propagated via tissue culture to generate F_1_ seedlings. In April 2005, these seedlings were subjected to field trials alongside 11 commercial cultivars, landrace and wild varieties. One F_1_ seedling derived from 1B exhibited superior traits, such as larger fruit size, high yield and enhanced fruit morphology. In August 2005, shoot tips were excised from this seedling for meristem tip culture, producing approximately 75 000 virus-free plantlets and establishing a clonal lineage. Subsequent varietal comparative trials and regional tests in April 2006 confirmed its consistent superiority to over ten commercial varieties in terms of comprehensive agronomic traits. This lineage was officially registered as “Yongqing No. 1” (Ma, Mo, & Bai, 2008).

#### 4.3.2 Hybrid selection of second-generation seedless polyploid varieties

Seeds account for 40%–50% of the fresh weight of diploid *S. grosvenorii*, but they do not contribute mogrosides. Excessive waste derived from seeds during mogroside extraction reduces yield efficiency and increases production costs. Developing seedless or low-seed polyploid varieties offers a fundamental solution to this issue.

Our team hybridized a high-mogroside V diploid cultivar (“Nongyuan B6”) with a naturally mutated tetraploid male pollinator (“Yaoyuan Xiongbai No.1”). When the triploid F_1_ progeny were pollinated with standard Qingpiguo varieties, they produced nearly seedless fruits. Through single-plant selection, an elite female line with minimal seeds, medium fruit size and abundant, fine-textured flesh was identified. Shoot tips from this line were propagated via tissue culture to establish a clonal variety. In 2008, stability trials and comparative assessments in germplasm nurseries confirmed the retention of its key traits, including seedlessness, flesh quality and mogroside V content, which are comparable to those of mainstream cultivars. This variety was formally registered as “Yaoyuan Seedless No. 1” (Mo et al., 2014).

### 4.4 Polyploid induction and stimulative parthenocarpy in *S. grosvenorii*

#### 4.4.1 Research on polyploid induction in S. grosvenorii

*S. grosvenorii* is naturally diploid. While spontaneous polyploidization occasionally occurs, its low mutation frequency and association with detrimental traits limits its breeding utility. Therefore, establishing efficient artificial chromosome doubling protocols for elite cultivars is critical for developing seedless varieties.

Research indicates that a technical system was developed for inducing chromosome doubling, which enabled the creation of seedless triploid lines. Preliminary field evaluations of triploid yield and quality traits revealed that triploids exhibited seedless fruit. However, compared to diploid controls, triploids showed reduced yield metrics, including smaller fruit equatorial diameter and lower fresh and dry weights. Quality indices (total mogrosides, mogroside V, water extract, total sugars, and total flavonoids) displayed inconsistent trends, with some components increasing and others decreasing (Dong, 2014). These variations may be correlated with differences in seedling vigor and prolonged growth cycles of triploids. Further evaluations, including clonal propagation of triploid lines and expanded hybrid combination trials, are required to assess the feasibility of breeding high mogroside V seedless varieties.

#### 4.4.2 Research on stimulative parthenocarpy in *S. grosvenorii*

To address labor-intensive nature of manual pollination and the low processing efficiency involved in *S. grosvenorii* cultivation, a protocol was developed for the induction of parthenocarpy (Tu, 2015). They investigated its effects on fruit development, quality parameters, phytohormone regulation, and molecular mechanisms. The results showed that parthenocarpic fruits exhibited normal developmental patterns and mogroside accumulation, with significantly reduced seed content. This innovation alleviates the challenges of seed-derived purification during mogroside extraction, thereby reducing production costs and eliminating the need for manual pollination.

## 5 Biosynthesis of mogrosides

### 5.1 Overview of biosynthesis pathway

Mogrosides belong to the cucurbitane-type triterpenoid saponins. Their biosynthesis process is similar to that of other triterpenoid saponins. It begins with the isoprenoid pathway, where squalene is cyclized by the action of squalene epoxidase to form the triterpene skeleton. This skeleton undergoes chemical modifications such as oxidation, substitution, and glycosylation mediated by cytochrome P450 (CYP450)-dependent monooxygenases, uridine diphosphate (UDP)-dependent-glycosyltransferases (UGTs), and other enzymes, ultimately forming different triterpene aglycones and corresponding glycoside end products (Xiong et al., 2011, Sun et al., 2020).

Mogroside V is a cucurbitane-type triterpene saponin product found in the cytoplasm. Its precursors, isopentenyl pyrophosphate (IPP) and dimethylallyl pyrophosphate (DMAPP), are generated from acetyl-CoA via the mevalonate (MVA) pathway (Chappell, 1995). First, two molecules of acetyl-CoA are converted into 3-hydroxy-3-methylglutaryl-CoA (HMG-CoA) by the action of acetoacetyl CoA thiolase (AACT) and 3-hydroxy-3-methylglutaryl-CoA synthase (HMGS). Subsequently, HMG-CoA is catalyzed by 3-hydroxy-3-methylglutaryl-CoA reductase (HMGR) to form MVA. Then, through the action of mevalonate kinase (MK), mevalonate diphosphate kinase (PMK), and mevalonate decarboxylase (MVD), mevalonate phosphate (MVP), mevalonate pyrophosphate (MVPP), and IPP are formed, successively. IPP is then converted into its double-bond isomer DMAPP by isopentenyl pyrophosphate isomerase (IPI) (Nagegowda, & Gupta, 2020).

Second, geranyl pyrophosphate synthase (GPS) catalyzes the formation of geranyl pyrophosphate (GPP) from IPP and DMAPP. Then, farnesyl pyrophosphate (FPP) is formed by IPP and GPP under the catalysis of farnesyl pyrophosphate synthase (FPS). FPP is converted into squalene (SQ) under the catalysis of squalene synthase (SQS). SQS is a bifunctional enzyme that first catalyzes the condensation of two molecules of FPP to form presqualene diphosphate (PSPP), and then, in the presence of nicotinamide adenine dinucleotide phosphate (NADPH) and Mg^2+^, converts PSPP into SQ (Harrison, 1988). SQ undergoes two consecutive epoxidation reactions under the action of squalene epoxidase (SQE), producing 2,3-epoxysqualene and 2,3;22,23-diepoxysqualene, the latter of which is cyclized into 24,25-epoxy cucurbitadienol by cucurbitadienol synthase (CDS) (Itkin et al., 2016). Notably, the known triterpene frameworks of oleanane-type, dammarane-type, and tirucallane-type are almost exclusively formed from 2,3-epoxysqualene as a substrate, which is then cyclized by different oxidosqualene cyclases (OSCs) followed by further chemical modifications such as oxidation, substitution, and glycosylation (Moses, Pollier, Thevelein, & Goossens, 2013). Early chemical studies have found that the key cyclization product cucurbitadienol is present in *S. grosvenorii*, suggesting that CDS may directly catalyze the cyclization of 2,3-epoxysqualene into the scaffold substance cucurbitadienol (Shibuya et al., 2004, Ukiya et al., 2002). This then undergoes a series of oxidations by CYP450 enzymes to produce mogrol, although this pathway has yet to be validated.

**Fig. 3:**
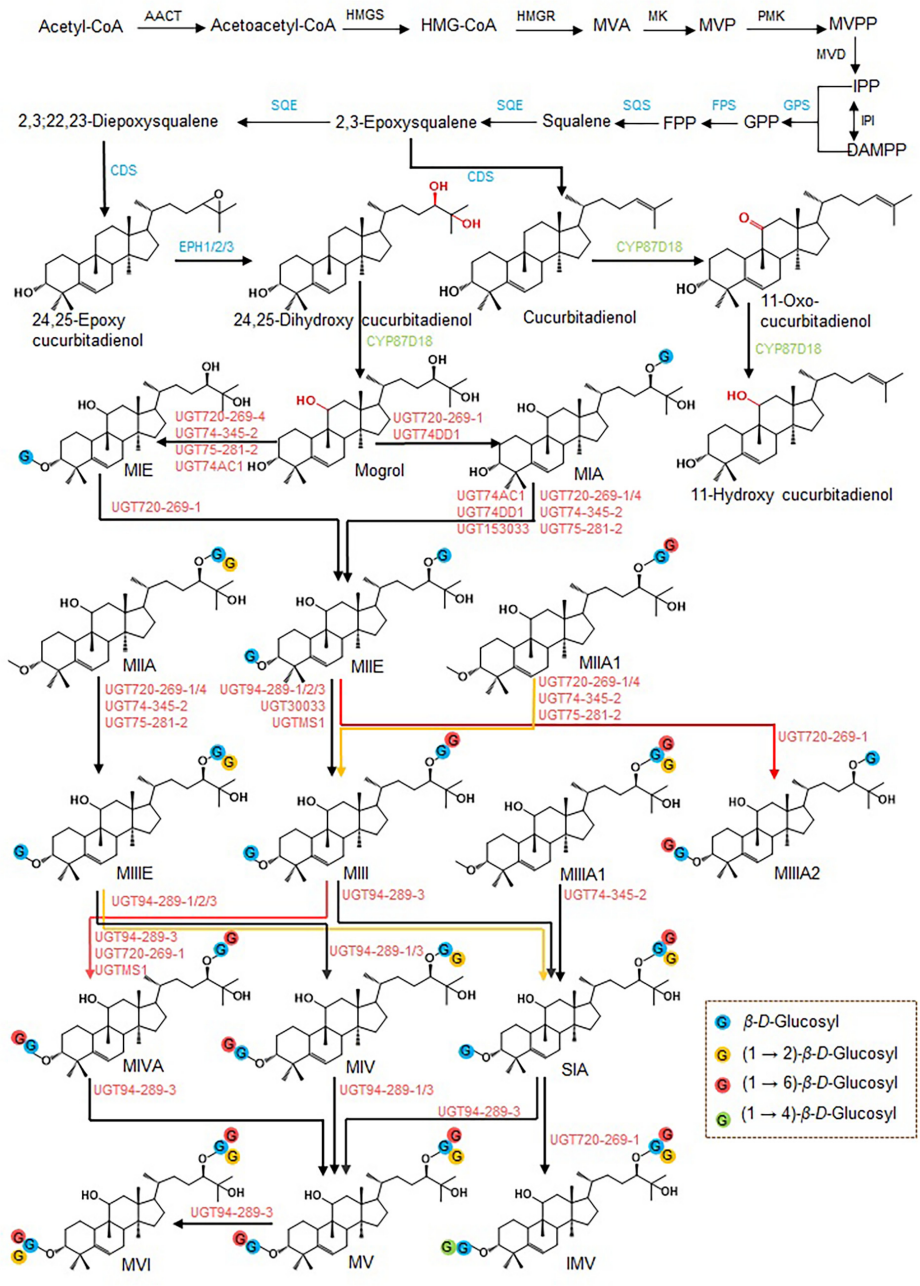
Biosynthetic pathway of mogrosides in *S. grosvenorii*. MIE: mogroside IE, MIA: mogroside IA, MIIE: mogroside IIE, MIIA: mogroside IIA, MIIA1: mogroside IIA1, MIII: mogroside III, MIIIE: mogroside IIIE, MIIIA1: mogroside IIIA1, MIIIA2: mogroside IIIA2, SIA: siamenoside I, MIV: mogroside IV, MIVA: mogroside IVA, MIVX: mogroside IVX, MV: mogroside V, IMV: isomogroside V, MVI: mogroside VI. The arrows of different colors are only for the convenience of distinguishing the crisscrossing routes.

Finally, 24,25-epoxy cucurbitadienol undergoes hydroxylation at the C_24_ and C_25_ positions under the action of epoxide hydrolase (EPH), resulting in *trans*-24,25-dihydroxyl cucurbitadienol. This compound is then hydroxylated at the C_11_ position by SgCYP87D18, a member of the CYP450 family, to produce the aglycone mogrol. UGTs family member SgUGT720-269–1, sequentially glycosylates the C_24_ and C_3_ positions of mogrol, yielding mogroside IA1 and mogroside IIE. Mogroside IIE is glycosylated at the C_24_ position by SgUGT94-289–3, yielding mogroside III. Mogroside III is then branched glycosylated at the C_3_ and C_24_ positions by SgUGT94-289–3, resulting in the formation of a trisaccharide product mogroside IVA and a tetrasaccharide product, siamenoside I, which are further glycosylated to ultimately produce the primary sweet component of *S. grosvenorii*, mogroside V (Fig. 3) (Itkin et al., 2016, Zhang et al., 2016, Dai et al., 2015, Lu et al., 2024, Gong et al., 2024, Li et al., 2022).

### 5.2 Cloning and characterization of the key mogroside biosynthesis genes

RNA-seq and digital gene expression profile (DGE) analysis were conducted in fruits at 3 d, 50 d, and 70 d post-pollination (Tang et al., 2011). Then identified and rapid-amplification of cDNA ends (RACE)-cloned 31 full-length candidate genes involved in the metabolic synthesis of mogrosides, including upstream precursor synthesis-related MVA pathway genes *SgAACT*, *SgHMGS*, *SgHMGR*, *SgMK*, *SgPMK*, and *SgMVD.* Additionally, genes related to the synthesis of the skeleton compound 24,25-epoxy cucurbitadienol, such as *SgIPI*, *SgGPS*, *SgFPS*, *SgSQS*, and *SgCDS*, as well as downstream branch pathway genes for specific modifications like CYP450s and UGTs. In terms of functional gene validation related to the metabolic synthesis pathway of mogroside V, genes *SgHMGR*, *SgSQS*, *SgSQE*, *SgCDS*, and several CYP450s, EPHs, and UGTs have been validated through prokaryotic expression in *Escherichia coli* or eukaryotic expression in *Saccharomyces cerevisiae* (yeast) (Xing et al., 2013, Tu, 2015; Zhao et al., 2018a; Zhao et al., 2017).

#### 5.2.1 Squalene epoxidase

Squalene epoxidase (SQE) can catalyze the double epoxidation reaction of squalene in many triterpene synthase systems, simultaneously producing mono- and di-epoxides simultaneously, specifically 2,3-epoxysqualene and 2,3;22,23-diepoxysqualene (Rasbery et al., 2007, Nelson et al., 1981, Suzuki et al., 2002). In 2018, two full-length sequences annotated as *SQE* genes cloned from *S. grosvenorii*, both containing a complete open reading frame (ORF) of 1 575 bp, encoding 524 amino acids, named *SgSQE1* and *SgSQE2*, respectively. Molecular docking of protein substrates indicated that two SgSQEs could interact with the ligand 2,3-epoxysqualene to form hydrogen bonds, suggesting a potential function in producing diepoxysqualene (Zhao et al., 2017). Prior to this, it has been confirmed that SgSQE can perform double epoxidation to synthesize the key mogroside substrate, 2,3; 22,23-diepoxysqualene (Itkin et al., 2016).

#### 5.2.2 Cucurbitadienol synthase

Cucurbitadienol synthase (CDS), a member of the OSC family, is a key cyclization enzyme in the synthesis of mogrosides. *SgCDS* gene RACE cloned from *S. grosvenorii*, obtaining a full-length ORF of 2 280 bp (Zhao, 2014). A yeast expression vector was constructed, and after transformation into yeast strain IVF and induction, the vector was able to synthesize cucurbitadienol using the yeast’s own 2,3-epoxysqualene substrate, thereby confirming the function of SgCDS for the first time. Subsequently, *SgCDS* was also identified through RNA sequencing (RNA-seq) and digital gene expression profiling (DGE) analysis from *S. grosvenorii*. Its full-length cDNA is 2 800 bp long and encodes a protein comprising 759 amino acids. (Dai et al., 2015). The cyclization function of SgCDS was validated using yeast strain GIL77, which can cyclize 2,3-epoxysqualene into cucurbitadienol. These results led researchers to speculate that the synthesis of mogrol was formed by a series of CYP450s oxidations of cucurbitadienol, although this metabolic pathway had not been verified. It wasn't until the research reports indicated a functional analysis of SgCDS had been conducted using brewer’s yeast and transformed tobacco, that they discovered SgCDS could not only cyclize 2,3-epoxysqualene to produce cucurbitadienol but also cyclize 2,3;22,23-diepoxysqualene to generate 24,25-epoxycucurbitadienol (Itkin et al., 2016). After validation through downstream functional enzymes, they confirmed that 24,25-epoxycucurbitadienol is a substrate for the synthesis of mogrosides.

#### 5.2.3 Epoxide hydrolase

Epoxide hydrolase (EPH) is a widely distributed enzyme that catalyzes the hydrolysis of epoxides to form the corresponding vicinal diols (Barth, Fischer, Schmid, & Pleiss, 2004). Over 100 epoxide hydrolases have been identified or predicted, but research on this type of enzyme in relation to plant secondary metabolism is still relatively scarce. Over 40 CYP450s were screened, expressed during the young fruit stage of *S. grosvenorii* through genomic and transcriptomic data for functional expression in yeast systems producing cucurbitadienol, but no candidate genes capable of hydroxylating C_24_ or C_25_ of cucurbitadienol were observed (Itkin et al., 2016). However, three epoxide hydrolases-*SgEPH1*, *SgEPH2*, and *SgEPH3*-when expressed individually in GIL77 yeast containing *SgCDS*, showed approximately threefold increases in the levels of 24,25-dihydroxyl cucurbitadienol compared to yeast containing only *SgCDS*. This confirmed that EPH is involved in the synthesis of mogrol, catalyzing 24,25-epoxycucurbitadienol to *trans*-24,25-dihydroxyl cucurbitadienol. This study challenged the initial hypothesis that CYP450 enzymes were responsible for all hydroxylation processes leading to the formation of mogrol.

#### 5.2.4 CYP450 and cytochrome P450 reductase

CYP450 is a supergene family in plants that plays a key role in the oxidative reactions involved in the synthesis of natural products, including terpenes, flavonoids, alkaloids, and lignin (Zhang et al., 2023, Chakraborty et al., 2023). Seven candidate CYP450 genes were identified and screened, involved in the synthesis of mogrosides based on RNA-seq and DGE (Tang et al., 2011). Further demonstrated that CYP87D18 (also known as CYP102801) has the activity to catalyze the oxidation of cucurbitadienol at the C11 position to produce 11-oxo cucurbitadienol and 11-hydroxy cucurbitadienol (Zhang et al., 2016). When co-expressed with *SgCDS* in yeast, this enzyme can also produce 11-oxo-24,25-epoxy cucurbitadienol. Almost simultaneously, 40 *CYP450s* expressed in developing fruits were functionally validated in *E. coli* and *S. cerevisiae* (Itkin et al., 2016). The results showed that CYP102801 (CYP87D18) catalyzes the hydroxylation of *trans*-24,25-dihydroxy cucurbitadienol at the C11 position to produce mogrol. *CYP450* genes require the presence of the partner molecule CYP450 reductase (CPR) for their activity. Another experiment discovered that two genes annotated as CPR through transcriptome sequencing and cloned two full-length sequences of 2 124 bp, which encode 707 amino acids. To verify the role of CPR, the SgCPR1 and SgCPR2 full-length sequences were linked to the Gal10 promoter-driven yeast plasmid expression vector pEsc-Trp and transformed into the yeast strain YJ14 along with a vector containing SmCYP716AH1 (a known functional *CYP450* gene from *Salvia miltiorrhiza* Bge.) (Zhao et al., 2018b). Ultra performance liquid chromatography (UPLC) detection results indicated that SmCYP716AH1 could catalyze the production of ferruginol from miltiradiene in the presence of either SgCPR1 or SgCPR2, confirming that both SgCPR1 and SgCPR2 have the function of NADPH-CYP450 reductase.

#### 5.2.5 UDP glycosyltransferase (UGT)

UGTs are key terminal modifying enzymes in the synthesis of mogrosides and are part of a supergene family in plants. Several UGTs from *S. grosvenorii* have been functionally validated. Based on transcriptome sequencing combined with changes in the content of mogrosides, a total of eight candidate unigenes that may be involved in the glycosylation to produce mogroside V were screened and RACE-cloned, six of which have been submitted to the GenBank database: SgUGT1 (HQ259620.1), SgUGT2 (HQ259621.1), SgUGT3 (HQ259622.1), SgUGT4 (HQ259623.1), SgUGT6 (HQ259626.1), and SgUGT7 (HQ259625.1) (Tang et al., 2011). The eight UGT genes were then transformed into yeast for functional validation, and it was found that SgUGT1(SgUGT720-269-1) can catalyze substrate mogrol to mogroside IA (Ma & Mo, 2024). UGT74AC1 was identified in *S. grosvenorii*. *In vitro* enzyme activity assays showed that UGT74AC1 can specifically transfer a glucose moiety to the C_3_ position of mogrol, forming mogroside IE (Dai et al., 2015). A total of 131 SgUGTs were identified, and 100 of these were screened for functional validation in *E. coli* and *S. cerevisiae* (Itkin et al., 2016). The results indicated that UGT74-345-2, UGT75-281-2, UGT720-269-1, and UGT720-269-4 are responsible for primary glycosylation at the C_3_ position, with UGT720-269-1 being the only enzyme responsible for primary glycosylation at the C_24_ position. UGT720-269-1, UGT94-289-1, UGT94-289-2, and UGT94-289-3 are responsible for the branched glycosylation of glucose chains at C_3_ and C_24_. Two UGTs (UGT153033 and UGT30033) catalyze the formation of glycosidic linkages at C_3_ and/or C_24_ of mogrosides have been screened and verified (Lu et al., 2024). Using mogroside IA1 as the sugar acceptor, the catalytic activity of UGT153033 was tested, and it was found that UGT153033 could completely convert mogroside IA1 to mogroside IIE without any byproducts. UGT30033 can use mogroside IIE as the sugar acceptor to further catalyze C_24_ branched glycosylation to form mogroside III. A recent study reported a novel UGT (UGT74DD1) from *S. grosvenorii* that catalyzes the mogrol to mogroside IIE. After redesigning UGT74DD1, a mutant capable of catalyzing the mogrol to mogroside III was obtained (Gong et al., 2024). Transcriptomic analysis was conducted on *S. grosvenorii* at different growth stages and identified 40 candidate UGT genes (Li, et al., 2022). Sequence alignment showed that UGTMS1 (has 21 amino acids deficiency in *N*-terminal compared with UGT94-289–3) clusters with UGT94-289–3 in the UGT94 family, suggesting they may have similar glycosylation activities. Further *in vitro* activity assays demonstrated that UGTMS1 can catalyze the mogroside IIE to mogroside IIIA, and further glycosylation to form mogroside IVA with a *β*-(1–6) glycosidic bond. When using mogroside IIIE and mogroside IIIA as substrates, products mogroside IVA and mogroside IVE can be obtained.

### 5.3 Accumulation pattern and the structure-sweetness relationship of mogrosides

Mogrosides share a common aglycone, mogrol. Under the catalysis of UGTs, glycoses are sequentially added to gradually form sweet-tasting tetrasaccharides and pentasaccharides, with sweetness related to the number of glycoses and their linkages (Kasai et al., 1988, Li et al., 2014). The dynamic accumulation pattern of mogrosides in the fruit effectively explains the formation process of these compounds. As the fruit grows and develops, the composition of mogrosides changes dynamically (Tang et al., 2011, Li et al., 2007). The first 40 d after the pollination, the fruit mainly contains the bitter mogroside IIE and mogroside III. Between 40 and 50 d, the levels of mogroside IIE and mogroside III gradually decrease, while the sweet mogroside IV and mogroside V begin to accumulate. From 50 to 70 d, mogroside V rapidly accumulates, while mogroside IIE, mogroside III, and mogroside IV decrease and disappear. After 70 d, the fruit primarily contains sweet mogroside V, along with mogroside VI and mogroside VII, but the accumulation increases slowly.

The study on the structure-taste relationship of mogrosides indicates that the number of glucose units, the oxygen at position C_11_ of the mogrol, the way the glucose moieties are connected, and the hydroxylation of the side chain are related to taste perception (Kasai, 2008, Kasai et al., 1988, Li et al., 2014). Notably, the presence of at least four glucose units in the molecule is essential for taste generation. For example, compounds Mogroside IE, Mogroside IIE, and Mogroside III are bitter or tasteless, while Mogroside IV and Mogroside V are sweet. The *α*-hydroxylated compounds at position 11 taste sweet, whereas the *β*-hydroxyl series are tasteless, and the 11-oxo compounds, as well as the dehydro derivatives, taste bitter. Additionally, the distribution of glucose units is noteworthy in relation to sweetness. Siamenoside I, which has four glucose units, is currently the sweetest compound isolated in this type of glycoside, with a sweetness 563 times that of 5% sucrose, while its isomers Mogroside IVA and IV have sweetness levels of 300 and 392, respectively, despite having the same number of glucose units (Matsumoto, Kasai, Ohtani, & Tanaka, 1990).

## 6 Synthetic biology of mogrosides

**Fig. 4:**
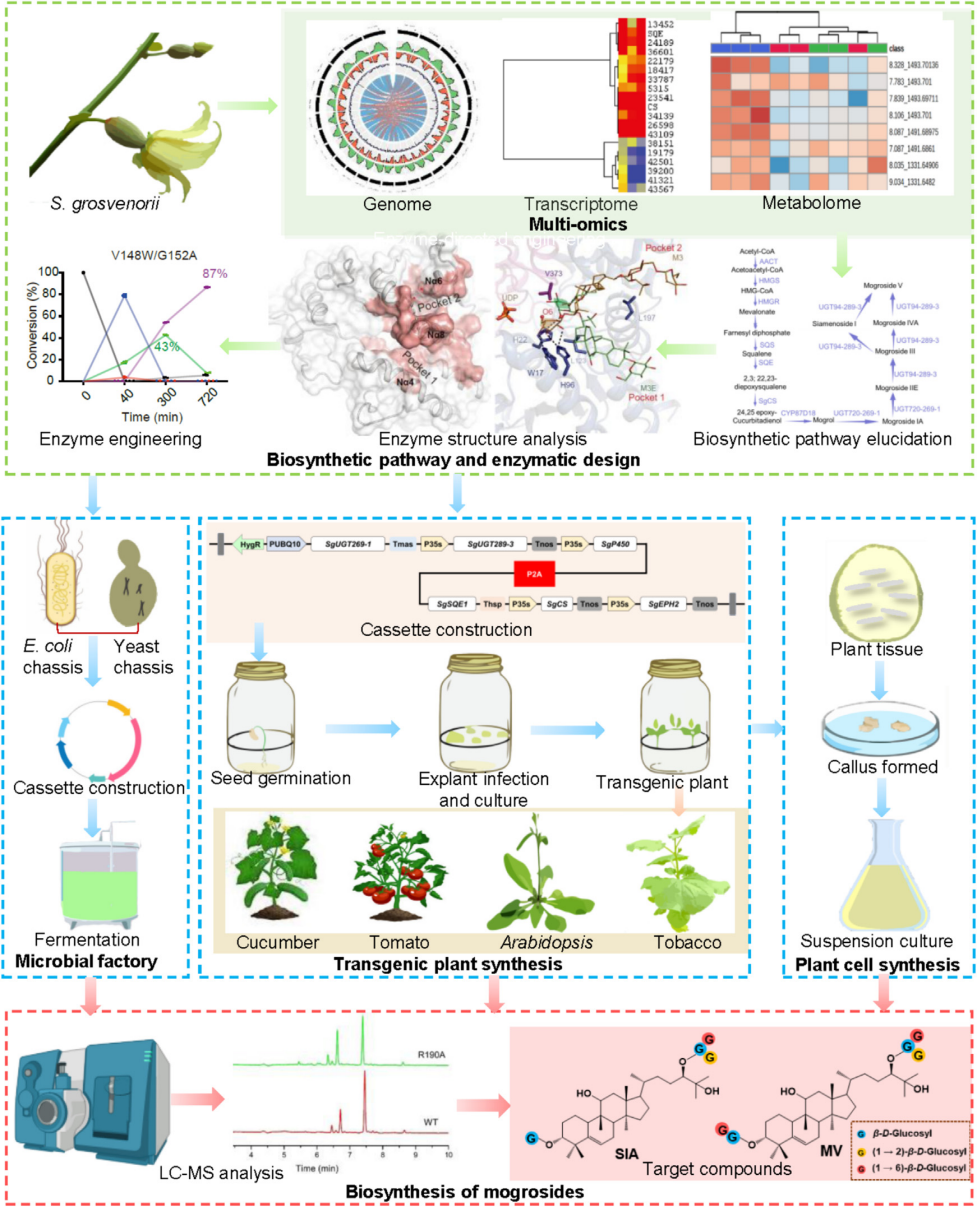
Sustainable mogroside production via integrated multi-omics and metabolic engineering.

Mogroside V is the main bioactive compound in *S. grosvenorii*, with a sweetness approximately 378 times that of 5% sucrose. It is non-calorie and non-toxic, making it an ideal natural sweetener for patients with diabetes and obesity to replace sucrose. However, mogroside V is present in low content in *S. grosvenorii*, and the cost directly extracting it from raw *S. grosvenorii* is high. Synthetic biology offers unique advantages for the efficient and sustainable acquisition of plant-derived natural products and has been applied to the synthesis of various natural products (Chen et al., 2012; Liu et al., 2021b; Liu et al., 2024). Therefore, research on synthetic biology provides an effective approach to realize large-scale heterologous biosynthesis and production of mogroside V. Currently, researchers both domestically and internationally have conducted studies on the biosynthesis of mogrosides using microbial heterologous synthesis with *E. coli* and *S. cerevisiae* as chassis, as well as plant-based heterologous synthesis (Fig. 4). Additionally, the suspension cell culture method can increase the yield of the target product, mogroside V, by artificially controlling the culture environment, and experimental results have demonstrated that it is a highly promising method for industrial production. As demonstrated in Fig. 4, an integrative multi-omics approach, including genome, transcriptome, and metabolome analyses, offers essential genetic insights for identifying genes linked to unexplored biosynthetic pathways. To optimize the use of natural enzymes, methods such as enzyme structure analysis and directed evolution are utilized to enhance their catalytic efficiency, substrate specificity, and regiospecificity (Li et al., 2020b; Cui et al., 2024). Various chassis, including bacteria, yeasts, and plants, can be engineered for metabolic pathway reconstruction and the sustainable production of mogrosides (Liao et al., 2023, Liao et al., 2022).

### 6.1 Microbial cell factories

Before the complete synthesis pathway of mogrosides was elucidated, researchers began synthetic biology studies targeting cucurbitadienol as the desired product. Our research group constructed a yeast expression vector pYES2-SgCDS and transformed it into the yeast strain IVF, resulting in a positive engineered strain that produces cucurbitadienol. This successfully established a new pathway for the biosynthesis of cucurbitadienol through yeast (Zhao, 2014). Subsequently, the methods and conditions for the biosynthesis of cucurbitadienol based on the yeast expression system were further optimized (Qiao et al., 2019). By overexpressing the global regulatory factor *UPC2* for triterpene synthase and knocking out the *ERG7* gene, the yield of cucurbitadienol was significantly increased from 7.80 to 61.80 mg/L. Additionally, the previously obtained yeast strain WD-2091 (which overexpresses the MVA pathway and *FPS*, *SQS*, *SQE*) was utilized to heterologously express and ferment the cloned *SgCDS*, achieving a yield of 27.44 mg/L for cucurbitadienol (Li et al., 2017). Further, by transferring the *SgCDS* gene from the high-copy plasmid pRS425 to the low-copy plasmid pRS313 to regulate *SgCDS* gene expression, obtaining engineering yeast 313-SL-CB, which improved the yield of cucurbitadienol by 202.07%, reaching a production of 1 724.10 mg/L through high-density fermentation. Recently, ahigh-level production of cucurbitadienol was achieved in *S. cerevisiae*, reaching a yield of 6.1 g/L through modular metabolic engineering and fermentation optimization. This is the highest yield reported for microbial synthesis of cucurbitadienol to date (Yin et al., 2025). Although the pathway of cucurbitadienol as a substrate for the synthesis of mogrosides has yet to be confirmed, the significant progress made in the biosynthesis research of cucurbitadienol holds important scientific value for the biosynthesis studies of known cucurbitacin compounds.

Different kinds of mogrosides are derived from mogrol through glycosylation modifications. The synthesis pathway of mogrol has already been constructed in *S. cerevisiae*. The yeast strain BY4743_YHR072 was used to co-express *SgCDS*, *SgEPH3*, *SgCYP87D18* and *AtCPR*, while incorporating the lanosterol synthase inhibitor R0 48–8072 to the culture medium (Itkin et al., 2016). The results indicated that the addition of the inhibitor cause to the accumulation of epoxide and 11-OH intermediates within 24 h, and mogrol was synthesized after 48 h of culture. According to research reports, a modular engineering strategy can be used to increase mogrol yield in yeast chassis cells. First, they constructed a *de novo* synthesis pathway for mogrol in *Saccharomyces cerevisiae*. Then, they systematically optimized the metabolic flux of each synthesis module in the mogrol metabolism, including enhancing precursor supply, utilizing a clustered regularly interspaced short palindromic repeats interference (CRISPRi) system to inhibit the sterol synthesis pathway, and optimizing the expression and reduction system of CYP450 enzymes. Ultimately, the titers of mogrol were increased to 9.1 μg/L (Wang et al., 2022).

Plant UGTs are a family of key enzymes that catalyze the glycosylation modification of plant natural products, conferring unique physiological activities and medicinal value. However, these UGTs usually have problems such as low catalytic efficiency and diversity of substrates. To address this, studies have focused on *S. grosvenorii* glycosyltransferase UGT74AC1. This has involved analyzing its crystal structure, performing directed evolution and carrying out sequence/structure engineering. Their efforts yielded a variant (UGTMG1) with dramatically enhanced catalytic efficiency: 4.17 × 10^4^-fold higher for mogrol and 1.53 × 10^4^-fold for UDP-glucose (UDPG), respectively (Li et al., 2020b). Subsequently, this team utilized the efficient variant of UGT74AC1 (UGTMG1), the mutant of UGTMS1 (UGTMS1-M7), and OsUGT91C1 (UGTMS2) to design an artificial polysaccharide glycosylation pathway (Li, et al., 2022). The glycosylated products were analysed by high-performance liquid chromatography-mass spectrometry (HPLC-MS) and the results indicate that mogrol have successfully converted into three types of mogrosides *in vitro*, achieving a product yield of 91%−99%. The study also constructed an engineered yeast for the synthesis of sweet mogrosides, testing the synthesis efficiency of the engineered strain using mogrol as the substrate, with a conversion rate close to 100%, and successfully obtaining a concentration of 19.26 mg/L of sweet mogroside IVA. However, the concentration of the synthesized product mogroside V was relatively low. A novel multi-enzyme system has been developed that combines three UGTs (UGT153033 and UGT30033 from *S. grosvenorii*, and yojK1 from *Bacillus subtilis*) with UDPG regeneration. This avoids the need for expensive UDPG (Lu et al., 2024). *In vitro* enzyme activity analysis indicated that the bitter mogroside IA1 could be rapidly converted into sweet mogroside IVX through this new synthetic pathway. Moreover, under conditions without using of costly UDPG, the multi-enzyme system catalyzed the sweet mogroside V in immature *S. grosvenorii* extracts to exceed 95%. A *de novo* biosynthesis pathway for mogroside V in *S. cerevisiae* for the first time was successfully constructed and implemented various metabolic strategies, achieving mogroside V titers of 10.25 mg/L in shake flasks and 28.62 mg/L in a 5-liter bioreactor (Qu et al., 2025). SgUGT94-289–3, the key UGT for mogroside V synthesis, exhibits significant substrate promiscuity and catalytic region selectivity, resulting in various by-products (Cui et al., 2024). Therefore, studies on the crystal structure and catalytic mechanism of SgUGT94-289–3 discovered that its *N*-terminus has a dual-pocket structure. This allows the two structurally different reaction ends of mogrosides to be presentd from different pockets to the active site of the glucosylation reaction, enabling multiple glycosylations. Based on the guidance from the crystal structure, we designed a series of mutants, achieving one-pot production of mogroside V/SIA *in vitro*, with the mutant V148M/G152A yielding the highest mogroside V production (94%) and only 3% by-products.

Siamenoside I is the sweetest mogrosides isolated from *S. grosvenorii* to date. However, the content of siamenoside I in the fruit is very low, only about 10% that of mogroside V, which limits its use as a natural sweetener. Therefore, conducting research on its biosynthesis has important practical value. Different microorganisms were screened for their ability to selectively hydrolyze the glycosidic bond in mogroside V to convert it into siamenoside I (Wang et al., 2019). The results showed that *Dekkera bruxellensis* possesses a unique enzyme that selectively facilitates the conversion of mogroside V to mogroside I, providing a feasible method for producing siamenoside I. A new compound, *α*-siamenoside I, was biosynthesized using two screened cyclodextrin glycosyltransferases. The glucose unit was selectively linked to the 6-hydroxyl of the 24-*O*-*β*-glucosyl portion of mogroside IIIE via an *α*-1,6-glycosidic bond, achieving a maximum yield of 59.3% (Xu et al., 2021). Compared to mogroside IIIE, the sweetness of *α*-siamenoside I significantly increases (508 times sweeter than 5% sucrose), and it has a similar mouthfeel to siamenoside I and good stability. Subsequently, this team engineered the UGT94-289–2 from *S. grosvenorii* to semi-rationally create an improved biocatalyst, UGT-M2, for the single glucosylation of mogroside IIIE to produce siamenoside I (Xu et al., 2022). Subsequently, an engineered *E. coli* strain was constructed that combined UGT-M2 with a UDPG regeneration system to avoid the need for expensive UDPG in the production of siamenoside I. After optimization, high-purity siamenoside I (> 96.4%) was efficiently prepared from mogroside IIIE at a scale of 1 L, with a productivity of 29.78 g/(L·d^−1^) and a molar yield of 76.5%. A continuous bioreactor with immobilized *β*-glucosidase was established and introduced water extract of *S. grosvenorii,* by controlling the flow rate, enabling the large-scale production of siamenoside I and mogroside IV for industrial applications (Chen et al., 2022a).

In terms of improving taste, some people do not accept mogrosides due to a certain metallic aftertaste. A central composite design method was adopted and cyclodextrin glucosyltransferases (CGTases) from three different bacterial sources (*Paenibacillus macerans*, *Geobacillus* sp., and *Thermoanaerobacter* sp.) were used to optimize the glycosylation reaction of mogroside extracts (Muñoz-Labrador et al., 2021). After further glycosylation, the taste of mogrosides was effectively improved.

### 6.2 Plant chassis system

Compared to microbial chassis cells, plant chassis have more refined organ and cell structures, offering unique advantages in aspects such as plant enzyme expression, synthetic pathway adaptability, and product tolerance. This provides another option for the heterologous synthesis of mogroside V. An In-Fusion-based gene stacking strategy for transgene stacking was developed, along with a multi-gene vector incorporating six genes involved in mogroside biosynthesis: *SgSQE1*, *SgCDS*, *SgEPH2*, *SgCYP450*, *SgUGT269-1*, and *SgUGT289-3* (Liao et al., 2023, Liao et al., 2022). Using *Agrobacterium*-mediated transformation, these genes were introduced into four higher plants: cucumber, tomato, tobacco, and *Arabidopsis*. Gene expression and analysis of mogroside products showed that transgenic tobacco leaves produced a large amount of mogroside IIE and a small amount of mogroside III. The content of mogroside IIE reached 5.66 µg/g fresh weight (FW), while the accumulation of mogroside III was 0.25 µg/g FW. In the T1 generation of transgenic *Arabidopsis*, relatively high levels of mogroside III and siamenoside I were accumulated, with siamenoside I reaching 10.04 µg/g FW and mogroside III accumulating approximately 0.20 µg/g FW. Additionally, a small amount of mogroside V was found in the fruits of the transgenic cucumber, with a content of about 0.58 µg/g FW. Furthermore, mogrol, mogroside IA1, mogroside IIE, mogroside III, and siamenoside I were detected in both the fruits and leaves of transgenic cucumbers. A trace amount of mogroside III was found in transgenic tomatoes. This research is the first to achieve the synthesis of sweet compounds such as mogroside V, siamenoside I, and mogroside III in transgenic cucumber and tomato plants, providing new ideas for sweet breeding research in vegetable crops. Additionally, the synthesis of mogrosides such as siamenoside I, mogroside III, and mogroside IIE in transgenic *Arabidopsis* and tobacco plants opens new avenues for the development of multi-source mogrosides raw material plants, laying a theoretical foundation for the development of plant factories for mogrosides synthesis.

Recombinant genes and plant transformation technology were used to construct an expression cassette containing the coding sequences of the enzymes in mogroside biosynthesis pathway, including *SgCDS*, *SgSQE, SgEPH*, *SgCYP450*, and *SgUGT*, which were introduced into the plant genome. Transgenic plants of tobacco and watermelon were obtained. Ultra performance liquid chromatography coupled with time-of-flight mass spectrometry (UPLC-TOF-MS) analysis results showed that mogroside IIA was produced in the transgenic tobacco leaves. Additionally, the presence of mogroside IIE was detected in the fruits and seed coats of transgenic watermelon, indicating the successful biosynthesis and accumulation of mogroside IIE in transgenic watermelon (Hayes et al., 2024).

### 6.3 Cell suspension culture

Plant suspension cell culture is characterized by rapid propagation, large-scale cultivation, independence from natural conditions, and the ability to provide a large quantity of uniform plant cell cultures. It has become one of the effective methods for the large-scale production of natural compounds used in the pharmaceutical, food, beverage, and cosmetics industries (Efferth, 2019, Eibl et al., 2018). Currently, researchers have established a suspension culture system using *S. grosvenorii* callus suspension culture and optimized the process conditions, achieving suspension scale-up in a 5-liter reactor (Chen, 2018).

A suspension cell culture system was set up using callus induced from *S. grosvenorii* stem segments to study the growth of *S. grosvenorii* suspension cells and the accumulation and kinetic changes of mogrosides (Lu, Zeng, Shen, Song, & Duan, 2011). The results showed that after 21 d of cultivation, the biomass of *S. grosvenorii* suspension cells and the concentration of mogroside V reached their maximum values, with a cell biomass of 693.0 g/L fresh cell weight (FCW) and mogroside V accounting for 5.77% of the cell weight. A suspension culture system was established using embryonic callus from *S. grosvenorii* and optimized the process conditions. After adding inducers, the yield of mogroside V in the suspension cells reached 0.83 mg/g dry cell weight (DCW) (Chen, 2018). Recently, Indian researchers established a suspension culture system using callus from the fruit peel of *S. grosvenorii* (Partap et al., 2022). After optimizing the conditions, the contents of mogroside V and 11-oxo mogroside V in the resulting suspension cell liquid were 1.76 mg/g DCW and 0.68 mg/g DCW, respectively, with a cell biomass of 184.58 g/L FCW. This study also compared the callus induction effects of different *S. grosvenorii* tissues, revealing that the callus induction response from the fruit peel and stem exceeded 90%.

## 7 Discussion and prospect

*S. grosvenorii* is an important TCM, listed in the *Chinese Pharmacopoeia* and included in the catalog of medicinal foods, making it suitable for use by patients with diabetes and obesity. Mogrosides are the main active component of *S. grosvenorii* and are currently the most potent natural sweetener discovered. Modern pharmacological studies have shown that mogrosides possess various biological activities, such as immunomodulatory, hepatoprotective, hypoglycemic, anti-tumor, and antioxidant effects, indicating good potential for health product and pharmaceutical development (Liu et al., 2018, Li et al., 2022). Additionally, mogrosides have an extremely high sweetness level and are considered safe for human consumption, recognized as safe food additive in several countries. Therefore, mogrosides have significant application prospects in food additives, functional foods, and dual-purpose medicinal foods.

### 7.1 Cultivation factors affecting mogrosides content

#### 7.1.1 Variety selection

*S. grosvenorii* is a dioecious plant native to southern China. It exhibits high genetic diversity in both wild and cultivated germplasm and has evolved multiple ecotypes and superior variants (Li, Jiang, & Li, 2010). Despite genetic erosion, existing cultivated accessions still demonstrate substantial variation in total mogroside content (4.19%–10.20%) and mogroside V content (0.25%–1.94%), with an eight-fold difference between extreme accessions (Liu et al., 2007, Mo et al., 2008, Zhou et al., 2011). These traits are predominantly regulated by genetic factors, showing broad-sense heritability of 95.12% for total mogroside and 95.14% for mogroside V, with narrow-sense heritability reaching 77.73% and 70.80%, respectively (Luo, 2012). Although molecular genetic linkage maps and genome sequencing have been established, the challenge is that the existing combination of third-generation and second-generation sequencing has failed to assemble the genome to the chromosomal level (Itkin et al., 2016, Xia et al., 2018). Consequently, the genetic mechanisms underlying mogroside V biosynthesis evolution and inter-accession variation remain uncharacterized, hindering breeding efficiency for high mogroside V cultivars. Enhancing genome assembly quality combined with genome-wide association studies (GWAS) across diverse germplasm could overcome the limitations of traditional population construction, elucidate mogroside V biosynthetic evolution, and advance molecular breeding strategies and germplasm conservation.

#### 7.1.2 Environmental factors

*S. grosvenorii* has strict habitat requirements. Previous research has revealed a 50% variation in mogroside V levels across ecological regions, primarily due to differences in environmental factors. Photoperiod studies show that light wavelengths can significantly impact photosynthetic rates and plant development (Huang, Zhao, Fu, Tang, & Huang, 2008). Moderate shading (50%–70%) for 30−90 d after pollination enhances mogroside V accumulation (Huang et al., 2009, Zhai et al., 2013). Optimal growth occurs at 25–30 ℃ in cool-humid climates. Research reports indicated that the following key thermal parameters in the authentic production area (Longjiang Township, Yongfu County, Guilin) are: a monthly mean temperature of 21–30 ℃, the total temperature during activities at or above 10 °C should not exceed 6 000 °C (Wang, Li, & Xiong, 2005). Studies indicated that temperature-mediated differential expression of mogroside V biosynthesis genes causes seasonal variation in quality, with autumn fruits showing superior mogroside V content (Peng et al., 2024). Soil amendment studies have demonstrated that silicon fertilization (150 g/plant) increases fruit dry weight by 10.6% and mogroside V content by 16.1% (Kang et al., 2017), while the use of combined bio-organic fertilizers [1 kg of Type I (purchased from Hanming Biotechnology Co., Ltd) + 0.5 kg of Type III (purchased from Guangxi Fengshun Biotechnology Co., Ltd., contains amino acids and trace elements)] enhances yield by 10.34% and increases the content of mogrosides by 16.44% (Liang et al., 2019). These findings highlight the critical importance of light (shading), temperature (cool conditions) and soil nutrients (Si, N, P and K), particularly low temperature. The necessitates systematic studies on the association between temperature and mogroside V to clarify the regulatory mechanisms.

#### 7.1.3 Postharvest processing

Processing is critical in determining medicinal suitability. Huang observed the progressive degradation of bioactive components (mogroside V, water extracts, sugars and vitamin C) as drying temperatures increased (from 60 to 120 ℃) (Huang, Mo, Ma, Li, & Wang, 2009). Constant-temperature drying showed inferior retention compared to variable-temperature protocols, with mogroside V decreasing from 1.01% at 70°C to 0.23% at 120°C, the latter being below the 0.50% standard set by the *Chinese Pharmacopoeia*. Traditional 7–15 d of “saccharification” at room temperature facilitates mogroside V accumulation (Fang, Wang, Liu, & Xu, 2017). Various drying methods were compared and found that microwave-vacuum drying retained the highest level of mogroside V (1.63%), outperforming microwave drying (1.58%), vacuum drying (1.38%), far-infrared drying (1.21%) and convective drying (0.97%) (Liu, Deng, Ning, Zhou, & Wang, 2017). Current limitations in processing research include inadequate temperature control standardization, limited parameter optimization and unexplored molecular regulatory mechanisms, indicating substantial potential for process improvement through systematic parameterization and mechanistic studies.

### 7.2 Molecular breeding of *S. grosvenorii*

#### 7.2.1 Molecular marker-assisted breeding in S. grosvenorii

As a dioecious and allogamous plant, *S. grosvenorii* exhibits high heterozygosity (1.5%) and significant inbreeding depression. This makes pure-line breeding time-consuming and difficult. Fortunately, mature tissue culture and cutting propagation technologies enable backcross breeding of elite varieties as a viable strategy. To accelerate breeding progress, molecular markers are required for major-effect traits (e.g., flowering time, inflorescence branching, fruit morphology) to facilitate foreground and background selection in backcross progenies. This approach is particularly impactful for breeding elite male lines, as molecular markers can enhance the accuracy and efficiency of selection in the absence of fruit-based phenotypic evaluation. Furthermore, the lack of germplasm pedigree data and long-term indiscriminate manual pollination have exacerbated genetic admixture. Molecular marker-assisted selection will critically reduce parental selection ambiguity in *S. grosvenorii* breeding.

#### 7.2.2 Genetic engineering breeding in S. grosvenorii

Fruit raw materials account for around 70% of mogroside processing costs. A low mogroside content also increases extraction expenses, which restricts industry’s ability to scale up. Concurrently, viral and nematode diseases severely compromise yield, profitability, and product safety. Cultivating green, high-quality, and high-yield varieties is essential in order to address these challenges.

Gene editing breeding, characterized by precision, efficiency, and non-transgenic outcomes, represents a frontier in plant biotechnology (Gao, 2021). To overcome the limitations of traditional transgenic methods, such as laborious process of selecting non-transgenic elite plant selection, low transformation efficiency, and food safety concerns, it is essential to optimize leaf disc-induced callus regeneration systems, screen *Agrobacterium*-mediated transformation parameters, develop CRISPR plasmid vectors for *S. grosvenorii*. These innovations will establish an efficient, targeted and DNA-free gene editing system to break breeding bottlenecks and accelerate cultivar development, germplasm innovation, and molecular marker discovery. Notably, SgUGT94-289–3 mutants were generated with greatly improved mogroside V and siamenoside I production from mogroside IIE in an *in vitro* one pot setup (Cui, et al., 2024). Editing SgUGT289-3 could produce new breeding materials with a high mogroside V content.

#### 7.2.3 Molecular design breeding in S. grosvenorii

As a regionally specialized crop, the development of molecular design breeding for *S. grosvenorii* is currently hindered by insufficient foundational research and fragmented technological integration. Despite the rapid advancement of molecular design breeding in major crops, its application in *S. grosvenorii* remains underdeveloped. This requires systematic planning and prioritized investment in core research areas.

The following strategic framework is proposed to establish a robust molecular design breeding system: Firstly, mogroside biosynthesis genes with high activity should be integrated into a single cultivar using chromosome-level genome data and gene editing. Secondly, transcription factors, enhancers, promoters and activators should be screened and combined to optimize biosynthetic pathways. Thirdly, advanced crop breeding software and simulation tools should be adopted to establish a *S. grosvenorii*-specific molecular design framework.

### 7.3 Application of synthetic biology in *S. grosvenorii*

Currently, the primary method for obtaining mogrosides relies on *S. grosvenorii* extraction. However, the low content of mogrosides, along with a complicated extraction process and the slow growth rate of *S. grosvenorii* (easily affected by environmental disturbances) lead to low efficiency for large-scale production, making it difficult to meet the growing market demand. This leads to high prices for mogrosides, limiting their broad market application. Therefore, developing efficient new pathways for synthesizing mogrosides is crucial. In recent years, introducing the biosynthetic pathways of natural products into microbial cell factories for the efficient synthesis has become a research focus (Liu et al., 2021b). Using cell factories to produce mogrosides is one potential pathway for mass production, but it is still in its early stages and faces bottlenecks as high energy consumption and low yields, making it challenging to meet industrial production demands (Li, et al., 2022).

UGT-mediated glycosylation of mogrol is a key step in determining the sweetness of different mogrosides and contributes to the diversity of mogrosides. However, existing UGTs with catalytic activity for mogrosides have drawbacks such as low activity, poor stability, and low substrate selectivity. Currently, they cannot meet the specificity required for synthesizing products, making it difficult for heterologous microorganisms to achieve targeted high-purity synthesis of specific mogrosides, significantly limiting the application of such enzymes in the synthesis of mogrosides (Cui et al., 2024). Therefore, optimizing functional enzymes is crucial for achieving biosynthesis. Future research should focus on screening and identifying enzymes (especially UGTs) with high substrate selectivity and product specificity, or deeply analyzing the catalytic mechanisms of functional UGTs. Techniques such as site-directed mutagenesis or gene editing can be used to modify UGTs to enhance their characteristics.

The use of microbial cell factories for the biosynthesis of natural products is rapidly developing. Compared to traditional chemical synthesis, microbial cell factories have the significant advantage of reducing carbon emissions, but they still require the addition of exogenous glucose as a raw material, which differentiates them from truly green synthesis of specific natural products (Qu et al., 2025). The synthetic biology research of mogrosides began with microorganisms and is now expanding to more complex plant systems, particularly model plants such as *Arabidopsis* and tobacco, as well as vegetable crops like cucumber, watermelon and tomato, which can be developed as chassis cells for mogrosides production. Plant cells are generally easier to express plant-derived genes compared to commonly used activities engineering microbial cells, and their genetic manipulation systems are mature. They also offer various advantages, including short growth cycles and energy savings (Liao et al., 2023, Liao et al., 2022). With the increasing maturity of technologies such as multi-gene overexpression and gene editing, plant chassis heterologous synthesis is expected to develop into a new platform for the efficient production of mogrosides and other complex secondary metabolites. Furthermore, research on plant cell suspension culture technologies has advanced rapidly in recent years (Partap et al., 2022). Under the existing *S. grosvenorii* suspension cell culture system, targeted genetic and metabolic regulation based on the characteristics of the cells can be employed to modify the cells at the molecular level, promoting the synthesis of mogrosides and increasing yields. This approach holds promise for overcoming the technical bottlenecks associated with the low yields of mogrosides biosynthesis.

## 8 Conclusion

This review summarizes the multifaceted research on *S. grosvenorii*, placing it at the intersection of traditional use and modern biotechnology. The main obstacle to its wider commercial use-the low natural abundance of its key bioactive component, mogroside V-has been identified as a key challenge. Integrated analysis shows that overcoming this limitation requires a paradigm shift from conventional cultivation to precision breeding and synthetic biology. Advances in the functional characterization of biosynthetic genes have paved the way for molecular design breeding, providing a strategic approach to enhancing mogroside yields in plants. Meanwhile, the development of synthetic biology platforms that use microbial, plant and cell culture systems offers a disruptive alternative for the *de novo* production of mogrosides, independent of agricultural constraints. The way forward lies in the synergistic integration of these approaches. Future work should prioritize the identification and characterization of yet-unknown pathway enzymes, the optimization of chassis organisms, and the development of efficient genome-editing protocols for *S. grosvenorii*. Success in these areas will unlock the full commercial potential of *S. grosvenorii* as a source of natural sweeteners and serve as a model for improving other TCM plants facing similar challenges.

## CRediT authorship contribution statement

**Zuliang Luo:** Writing – original draft. **Yimei Zang:** Writing – original draft. **Jiaxian Su**: Data curation. **Qi Tang:** Project administration. **Limei Pan:** Resources. **Xiaojun Ma:** Supervision. **Chongnan Wang:** Writing – review & editing. **Changming Mo:** Writing – review & editing.

## Declaration of competing interest

The authors declare that they have no known competing financial interests or personal relationships that could have appeared to influence the work reported in this paper.
